# 4-Methyl­anilinium 2-carb­oxy­acetate

**DOI:** 10.1107/S1600536812024403

**Published:** 2012-06-02

**Authors:** Ying-Chun Wang

**Affiliations:** a College of Chemistry and Chemical Engineering, Southeast University, Nanjing 210096, People’s Republic of China

## Abstract

During the formation of the title salt, C_7_H_10_N^+^·C_3_H_3_O_4_^−^, an H atom of a carboxyl group was transferred to the amino group. All non-H atoms of the cation are essentially coplanar [r.m.s. deviation = 0.007 (4) Å]. The mean planes of the carboxyl­ate and carboxyl groups of the anion form a dihedral of 69.67 (1)°. In the crystal, N—H⋯O and O—H⋯O hydrogen bonds connect the anions and cations, forming a two-dimensional network parallel to the *bc* plane.

## Related literature

For the structures and properties of related compounds, see: Chen *et al.* (2001 ▶); Wang *et al.* (2002 ▶); Xue *et al.* (2002 ▶); Huang *et al.* (1999 ▶); Zhang *et al.* (2001 ▶); Ye *et al.* (2008 ▶). 
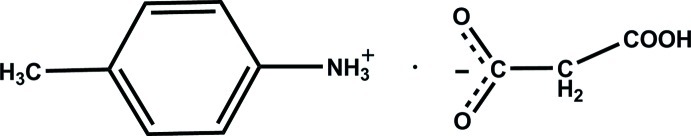


## Experimental

### 

#### Crystal data

C_7_H_10_N^+^·C_3_H_3_O_4_^−^
*M* *_r_* = 211.21
Monoclinic, 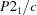

*a* = 12.7937 (19) Å
*b* = 9.2742 (16) Å
*c* = 8.5194 (17) Å
β = 104.853 (2)°
*V* = 977.1 (3) Å^3^
*Z* = 4
Mo *K*α radiation
μ = 0.11 mm^−1^
*T* = 123 K
0.10 × 0.05 × 0.05 mm

#### Data collection

Rigaku Mercury2 diffractometer
Absorption correction: multi-scan (*CrystalClear*; Rigaku, 2005 ▶) *T*_min_ = 0.910, *T*_max_ = 1.000
7787 measured reflections
2228 independent reflections
1943 reflections with *I* > 2σ(*I*)
*R*_int_ = 0.031

#### Refinement

*R*[*F* ^2^ > 2σ(*F* ^2^)] = 0.042
*wR*(*F* ^2^) = 0.111
*S* = 1.08
2228 reflections
138 parameters
4 restraints
H-atom parameters constrained
Δρ_max_ = 0.28 e Å^−3^
Δρ_min_ = −0.24 e Å^−3^

### 

Data collection: *CrystalClear* (Rigaku, 2005 ▶); cell refinement: *CrystalClear*; data reduction: *CrystalClear*; program(s) used to solve structure: *SHELXS97* (Sheldrick, 2008 ▶); program(s) used to refine structure: *SHELXL97* (Sheldrick, 2008 ▶); molecular graphics: *SHELXTL* (Sheldrick, 2008 ▶) and *DIAMOND* (Brandenburg, 1999 ▶); software used to prepare material for publication: *SHELXTL*.

## Supplementary Material

Crystal structure: contains datablock(s) I, global. DOI: 10.1107/S1600536812024403/lh5480sup1.cif

Structure factors: contains datablock(s) I. DOI: 10.1107/S1600536812024403/lh5480Isup2.hkl

Supplementary material file. DOI: 10.1107/S1600536812024403/lh5480Isup3.cml

Additional supplementary materials:  crystallographic information; 3D view; checkCIF report

## Figures and Tables

**Table 1:** Hydrogen-bond geometry (Å, °)

*D*—H⋯*A*	*D*—H	H⋯*A*	*D*⋯*A*	*D*—H⋯*A*
O1—H1⋯O4^i^	0.82	1.72	2.5353 (15)	175
N1—H1*A*⋯O2^ii^	0.91	1.94	2.8377 (17)	167
N1—H1*B*⋯O3^iii^	0.91	1.86	2.7642 (17)	171
N1—H1*C*⋯O1^iv^	0.91	2.21	2.8600 (17)	128
N1—H1*C*⋯O3^v^	0.91	2.32	2.9872 (17)	130
N1—H1*C*⋯O4^iv^	0.91	2.41	2.9655 (17)	120

Symmetry codes: (i) 
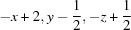
; (ii) 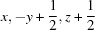; (iii) 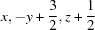; (iv) 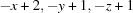; (v) 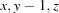.

**Fig. 1.**
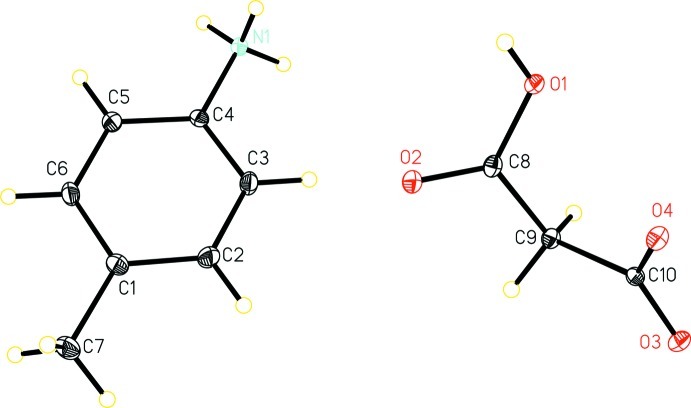
A view of the asymmetric unit with displacement ellipsoids drawn at the 30% probability level.

**Fig. 2.**
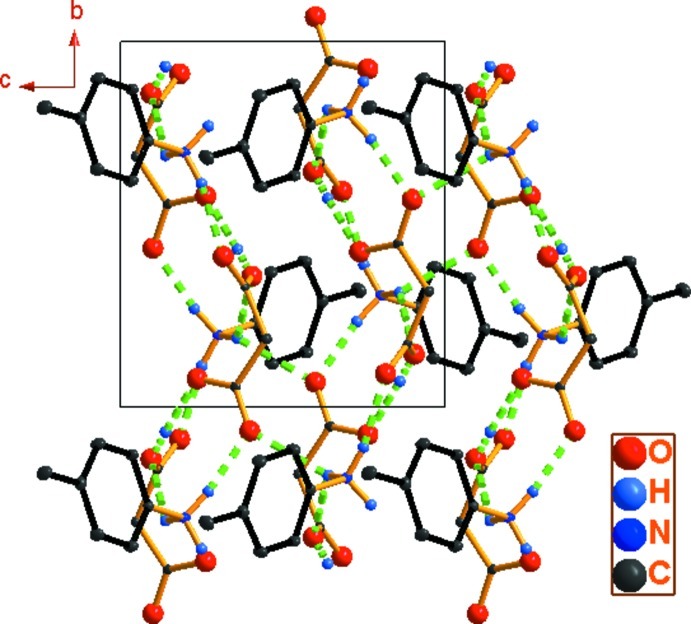
The crystal packing of the title compound viewed along the a axis showing hydrogen bonds as dashed lines.

## supplementary crystallographic information

### Comment

Simple organic salts containing strong intrermolecular H-bonds have attracted attention as materials which display ferroelectric-paraelectric phase transitions (Chen *et al.*, 2001; Huang, *et al.* 1999; Zhang, *et al.* 2001). With the purpose of obtaining phase transition crystals of organic salts, various organic molecules have been studied and a series of new crystal materials have been elaborated (Wang, *et al.* 2002; Xue, *et al.* 2002; Ye, *et al.* 2008). Herein, we present the synthesis and crystal structure of the title compound.

The molecular structure of the title salt is shown in Fig. 1. The asymmetric unit is composed of one 4-methylanilinium cation and one 2-carboxyacetate anion. All non-H atoms of the cation are essentially coplanar [r.m.s. deviation = 0.007 (4)Å ]. The mean planes of the carboxylate and carboxyl groups of the anion form a dihedral of 69.67 (1)°. In the crystal, N—H···O and O—H···O hydrogen bonds connect anions and cations to form a two-dimensional network parallel to the *bc*-plane (Fig. 2 and Table 1).

### Experimental

Malonic acid (10 mmol), 4-toluidine (10 mmol) and ethanol (50 mL) were added to a 100mL flask. The mixture was stirred at 333K for 2 h, and then the precipitate was filtrated off. Colourless crystals suitable for X-ray diffraction were obtained by slow evaporation of the solution.

### Refinement

H atoms attached to C atoms were placed in idealized positions and treated as riding with C–H = 0.95 Å (aromatic), C–H = 0.98 Å (methyl) and C–H = 0.99 Å (methylene) with *U_iso_*(H)=1.2*U_eq_*(C except methyl), *U_iso_*(H)=1.5*U_eq_*(C methyl). The positional parameters of the H atoms (N and O) were refined freely. And in the last stages of the refinement, they were restrained with H—N = 0.91 (2)Å and H—O = 0.82 (2)Å with *U_iso_*(H)=1.5*U_eq_*(N and O).

### Crystal data

**Table:**

C_7_H_10_N^+^·C_3_H_3_O_4_^−^	*F*(000) = 448
*M**_r_* = 211.21	*D*_x_ = 1.436 Mg m^−^^3^
Monoclinic, *P*2_1_/*c*	Mo *K*α radiation, λ = 0.71073 Å
Hall symbol: -P 2ybc	Cell parameters from 2228 reflections
*a* = 12.7937 (19) Å	θ = 2.8–27.5°
*b* = 9.2742 (16) Å	µ = 0.11 mm^−^^1^
*c* = 8.5194 (17) Å	*T* = 123 K
β = 104.853 (2)°	Block, colorless
*V* = 977.1 (3) Å^3^	0.10 × 0.05 × 0.05 mm
*Z* = 4	

### Data collection

**Table:**

Rigaku Mercury2 diffractometer	2228 independent reflections
Radiation source: fine-focus sealed tube	1943 reflections with *I* > 2σ(*I*)
Graphite monochromator	*R*_int_ = 0.031
Detector resolution: 13.6612 pixels mm^-1^	θ_max_ = 27.5°, θ_min_ = 2.8°
CCD profile fitting scans	*h* = −14→16
Absorption correction: multi-scan (*CrystalClear*; Rigaku, 2005)	*k* = −11→12
*T*_min_ = 0.910, *T*_max_ = 1.000	*l* = −11→11
7787 measured reflections	

### Refinement

**Table:**

Refinement on *F*^2^	Primary atom site location: structure-invariant direct methods
Least-squares matrix: full	Secondary atom site location: difference Fourier map
*R*[*F*^2^ > 2σ(*F*^2^)] = 0.042	Hydrogen site location: inferred from neighbouring sites
*wR*(*F*^2^) = 0.111	H-atom parameters constrained
*S* = 1.08	*w* = 1/[σ^2^(*F*_o_^2^) + (0.0567*P*)^2^ + 0.2948*P*] where *P* = (*F*_o_^2^ + 2*F*_c_^2^)/3
2228 reflections	(Δ/σ)_max_ < 0.001
138 parameters	Δρ_max_ = 0.28 e Å^−^^3^
4 restraints	Δρ_min_ = −0.24 e Å^−^^3^

### Special details

**Table:**

Geometry. All esds (except the esd in the dihedral angle between two l.s. planes) are estimated using the full covariance matrix. The cell esds are taken into account individually in the estimation of esds in distances, angles and torsion angles; correlations between esds in cell parameters are only used when they are defined by crystal symmetry. An approximate (isotropic) treatment of cell esds is used for estimating esds involving l.s. planes.
Refinement. Refinement of F^2^ against ALL reflections. The weighted R-factor wR and goodness of fit S are based on F^2^, conventional R-factors R are based on F, with F set to zero for negative F^2^. The threshold expression of F^2^ > 2sigma(F^2^) is used only for calculating R-factors(gt) etc. and is not relevant to the choice of reflections for refinement. R-factors based on F^2^ are statistically about twice as large as those based on F, and R- factors based on ALL data will be even larger.

### Fractional atomic coordinates and isotropic or equivalent isotropic displacement parameters (Å^2^)

**Table:**

	*x*	*y*	*z*	*U*_iso_*/*U*_eq_	
O1	1.00549 (8)	0.64322 (11)	0.40536 (12)	0.0154 (3)	
H1	1.0186	0.5694	0.3608	0.023*	
N1	0.83328 (10)	0.19556 (13)	0.69727 (15)	0.0149 (3)	
H1A	0.8293	0.1110	0.7495	0.022*	
H1B	0.8521	0.2678	0.7714	0.022*	
H1C	0.8839	0.1878	0.6397	0.022*	
C1	0.52657 (13)	0.28907 (18)	0.37780 (18)	0.0188 (3)	
O2	0.82906 (9)	0.58930 (12)	0.31709 (13)	0.0184 (3)	
C2	0.60240 (14)	0.39772 (18)	0.4318 (2)	0.0228 (4)	
H2A	0.5849	0.4941	0.3972	0.027*	
O3	0.87706 (9)	1.06665 (11)	0.39914 (12)	0.0164 (3)	
C3	0.70340 (13)	0.36805 (17)	0.53566 (19)	0.0199 (3)	
H3A	0.7546	0.4431	0.5709	0.024*	
O4	0.94461 (9)	0.92360 (11)	0.23717 (12)	0.0157 (3)	
C4	0.72805 (11)	0.22776 (16)	0.58657 (17)	0.0141 (3)	
C5	0.65550 (13)	0.11751 (17)	0.5333 (2)	0.0198 (3)	
H5A	0.6736	0.0212	0.5676	0.024*	
C6	0.55545 (13)	0.14905 (18)	0.42871 (19)	0.0209 (4)	
H6A	0.5056	0.0729	0.3911	0.025*	
C7	0.41642 (14)	0.31810 (19)	0.2661 (2)	0.0259 (4)	
H7A	0.3952	0.4177	0.2806	0.039*	
H7B	0.3635	0.2518	0.2920	0.039*	
H7C	0.4190	0.3036	0.1532	0.039*	
C8	0.90197 (12)	0.67134 (15)	0.38432 (16)	0.0135 (3)	
C9	0.88015 (12)	0.81514 (16)	0.45071 (17)	0.0139 (3)	
H9A	0.9251	0.8234	0.5637	0.017*	
H9B	0.8035	0.8181	0.4543	0.017*	
C10	0.90250 (11)	0.94594 (15)	0.35453 (16)	0.0126 (3)	

### Atomic displacement parameters (Å^2^)

**Table:**

	*U*^11^	*U*^22^	*U*^33^	*U*^12^	*U*^13^	*U*^23^
O1	0.0161 (5)	0.0117 (5)	0.0190 (5)	0.0001 (4)	0.0057 (4)	−0.0020 (4)
N1	0.0140 (6)	0.0135 (6)	0.0168 (6)	−0.0006 (5)	0.0033 (5)	0.0004 (5)
C1	0.0168 (8)	0.0219 (8)	0.0164 (7)	0.0007 (6)	0.0018 (6)	0.0005 (6)
O2	0.0168 (6)	0.0151 (6)	0.0217 (6)	−0.0021 (4)	0.0022 (4)	−0.0024 (4)
C2	0.0230 (8)	0.0161 (8)	0.0253 (8)	0.0018 (6)	−0.0012 (6)	0.0032 (6)
O3	0.0203 (6)	0.0123 (5)	0.0161 (5)	0.0022 (4)	0.0037 (4)	−0.0007 (4)
C3	0.0210 (8)	0.0156 (8)	0.0200 (7)	−0.0039 (6)	−0.0002 (6)	0.0000 (6)
O4	0.0206 (6)	0.0134 (5)	0.0143 (5)	−0.0004 (4)	0.0066 (4)	−0.0001 (4)
C4	0.0125 (7)	0.0166 (7)	0.0133 (6)	0.0011 (5)	0.0035 (5)	−0.0005 (5)
C5	0.0174 (8)	0.0156 (8)	0.0249 (8)	−0.0001 (6)	0.0024 (6)	0.0021 (6)
C6	0.0175 (8)	0.0181 (8)	0.0249 (8)	−0.0049 (6)	0.0014 (6)	0.0004 (6)
C7	0.0195 (8)	0.0264 (9)	0.0267 (8)	0.0004 (7)	−0.0036 (7)	0.0049 (7)
C8	0.0175 (7)	0.0126 (7)	0.0105 (6)	−0.0016 (5)	0.0039 (5)	0.0025 (5)
C9	0.0154 (7)	0.0134 (7)	0.0132 (6)	−0.0006 (5)	0.0044 (5)	0.0001 (5)
C10	0.0105 (6)	0.0126 (7)	0.0126 (6)	−0.0006 (5)	−0.0010 (5)	0.0000 (5)

### Geometric parameters (Å, º)

**Table:**

O1—C8	1.3162 (18)	C3—H3A	0.9500
O1—H1	0.8202	O4—C10	1.2684 (18)
N1—C4	1.4629 (18)	C4—C5	1.377 (2)
N1—H1A	0.9100	C5—C6	1.390 (2)
N1—H1B	0.9100	C5—H5A	0.9500
N1—H1C	0.9100	C6—H6A	0.9500
C1—C6	1.389 (2)	C7—H7A	0.9800
C1—C2	1.393 (2)	C7—H7B	0.9800
C1—C7	1.509 (2)	C7—H7C	0.9800
O2—C8	1.2260 (18)	C8—C9	1.502 (2)
C2—C3	1.393 (2)	C9—C10	1.531 (2)
C2—H2A	0.9500	C9—H9A	0.9900
O3—C10	1.2518 (18)	C9—H9B	0.9900
C3—C4	1.382 (2)		
			
C8—O1—H1	114.8	C1—C6—C5	121.63 (15)
C4—N1—H1A	109.5	C1—C6—H6A	119.2
C4—N1—H1B	109.5	C5—C6—H6A	119.2
H1A—N1—H1B	109.5	C1—C7—H7A	109.5
C4—N1—H1C	109.5	C1—C7—H7B	109.5
H1A—N1—H1C	109.5	H7A—C7—H7B	109.5
H1B—N1—H1C	109.5	C1—C7—H7C	109.5
C6—C1—C2	117.79 (14)	H7A—C7—H7C	109.5
C6—C1—C7	119.64 (14)	H7B—C7—H7C	109.5
C2—C1—C7	122.57 (15)	O2—C8—O1	124.00 (14)
C1—C2—C3	121.40 (15)	O2—C8—C9	122.29 (14)
C1—C2—H2A	119.3	O1—C8—C9	113.71 (12)
C3—C2—H2A	119.3	C8—C9—C10	115.06 (12)
C4—C3—C2	119.01 (15)	C8—C9—H9A	108.5
C4—C3—H3A	120.5	C10—C9—H9A	108.5
C2—C3—H3A	120.5	C8—C9—H9B	108.5
C5—C4—C3	121.01 (14)	C10—C9—H9B	108.5
C5—C4—N1	119.47 (13)	H9A—C9—H9B	107.5
C3—C4—N1	119.52 (13)	O3—C10—O4	125.55 (13)
C4—C5—C6	119.14 (14)	O3—C10—C9	116.57 (13)
C4—C5—H5A	120.4	O4—C10—C9	117.88 (12)
C6—C5—H5A	120.4		
			
C6—C1—C2—C3	−1.0 (3)	C2—C1—C6—C5	1.5 (2)
C7—C1—C2—C3	179.67 (16)	C7—C1—C6—C5	−179.12 (16)
C1—C2—C3—C4	−0.5 (3)	C4—C5—C6—C1	−0.6 (3)
C2—C3—C4—C5	1.5 (2)	O2—C8—C9—C10	−108.21 (16)
C2—C3—C4—N1	−179.19 (14)	O1—C8—C9—C10	71.74 (16)
C3—C4—C5—C6	−1.0 (2)	C8—C9—C10—O3	174.53 (12)
N1—C4—C5—C6	179.70 (14)	C8—C9—C10—O4	−5.34 (18)

### Hydrogen-bond geometry (Å, º)

**Table:**

*D*—H···*A*	*D*—H	H···*A*	*D*···*A*	*D*—H···*A*
O1—H1···O4^i^	0.82	1.72	2.5353 (15)	175
N1—H1*A*···O2^ii^	0.91	1.94	2.8377 (17)	167
N1—H1*B*···O3^iii^	0.91	1.86	2.7642 (17)	171
N1—H1*C*···O1^iv^	0.91	2.21	2.8600 (17)	128
N1—H1*C*···O3^v^	0.91	2.32	2.9872 (17)	130
N1—H1*C*···O4^iv^	0.91	2.41	2.9655 (17)	120

Symmetry codes: (i) −*x*+2, *y*−1/2, −*z*+1/2; (ii) *x*, −*y*+1/2, *z*+1/2; (iii) *x*, −*y*+3/2, *z*+1/2; (iv) −*x*+2, −*y*+1, −*z*+1; (v) *x*, *y*−1, *z*.
